# Nicorandil ameliorates neuropathic and inflammatory pain via TNF-α, IL6/MAPK_ERK1/2_ and NO/cGMP signaling

**DOI:** 10.1038/s41598-026-35272-4

**Published:** 2026-02-02

**Authors:** Rasha M. Badr, Salwa A. Abuiessa, Samar S. Elblehi, Elham A. Afify

**Affiliations:** 1 Department of Pharmacology and Toxicology, Faculty of Pharmacy, University of Alexandria, 1-el-Khartoum square-Azarita, Alexandria, Egypt; 2 Department of Pathology, Faculty of Veterinary Medicine, University of Alexandria, Alexandria, Egypt

**Keywords:** Neuropathic pain, Nicorandil, TNF-α, IL6, NO/cGMP, Diseases, Drug discovery, Medical research, Neuroscience

## Abstract

Recently, nicorandil exerted antinociception via TRPV1/opioid signaling. Herein, the entanglement of downstream signals and NO-cGMP-K_ATP_ pathway of nicorandil mediated antinociception was investigated against neuropathic pain induced by chronic constriction injury of sciatic nerve (CCI) and formalin evoked inflammatory pain. Nicorandil (150 mg/kg, twice, 2 h apart, PO) reversed mechanical and cold allodynia induced by CCI, reduced the licking time and number of flinches in formalin test. L-arginine (500 mg/kg, I.P), N(ω)-nitro-L-arginine methyl ester (L-NAME, 10 mg/kg, I.P), methylene blue (10 mg/kg, I.P), sildenafil (2.5 mg/kg, I.P) and glibenclamide (5 mg/kg, I.P) were tested 30 min before nicorandil. The inhibitory effect of nicorandil on mechanical and cold allodynia was partially attenuated by L-arginine, methylene blue and sildenafil. L-NAME but not glibenclamide, potentiated the antinociceptive action of nicorandil on cold allodynia. In formalin test, nicorandil reduced flinches; an effect that was partially reversed by L-arginine and sildenafil but not by L-NAME, methylene blue or glibenclamide. Nicorandil reduced serum levels of the oxidative marker (MDA) and the inflammatory mediators (TNF-α, IL-6 and COX-2). Immunohistochemical studies revealed that nicorandil blunted the elevation of MAPK_ERK1/2_ protein expressions in DRG whereas naloxone reversed that suppression. L-arginine and sildenafil reversed nicorandil mediated improvements of histopathological milieu of sciatic nerves and DRG. Taken together, these data demonstrate that nicorandil has an antiallodynic effect on neuropathic and inflammatory pain via inhibition of NO/cGMP pathways and reduction of oxidative stress and proinflammatory cytokines targeting ROS/TNF-α, IL6 /MAPK_ERK1/2_ signaling pathways. These findings highlight nicorandil’s potential as a promising multitarget therapeutic option for pain management through its anti-inflammatory and antioxidant properties.

## Introduction

Pain is described as unpleasant sensory and emotional experience caused by actual or potential tissue injury^1^. Neuropathic pain is a chronic devastating pain that affects 7–10% of general population^2,3^ and precipitates high economic burden^4^. Neuropathic pain is defined according to the International Association for the Study of Pain (IASP) as the pain that occurs as a consequence of a lesion or disease in the somatosensory system^5^. Neuropathic pain sufferers may experience intermittent or continuous spontaneous pain in addition to evoked pain. Evoked pain includes allodynia which is pain to non-painful stimuli and hyperalgesia which is exaggerated pain to normally noxious stimuli^6^.

Various mechanisms contribute to the initiation and maintenance of neuropathic pain^7^. Neuroinflammation, oxidative stress, ion channel alterations, central sensitization and dysfunction of the descending nociceptive modulatory system are among the suggested pathophysiological mechanisms of this debilitating pain^8^. Neuroinflammation and oxidative stress play a pivotal role in the pathogenesis of neuropathic pain. Therefore, targeting the inflammation and reactive oxygen species (ROS) is considered a promising therapeutic strategy for management of neuropathic pain^9^.

Nitric oxide (NO) plays a dual role in pain processing since it has both pronociceptive and antinociceptive actions^10^. Growing mass of evidence suggests that the NO/cGMP pathway within the spinal cord plays a role in the development of neuropathic pain. Numerous studies reported the activation of NO/cGMP pathway after peripheral nerve injury. Moreover, NO is thought to contribute to the processing of neuropathic pain via both NO/cGMP/PKG and NO/peroxynitrite pathways. Therefore, targeting the NO/cGMP pathway would be helpful in management of chronic neuropathic pain^11^.

Ion channel alterations is a crucial mechanism contributing to neuropathic pain processing^6^. Recently, Qian et al.^12^ reported that the decrease in ATP-sensitive potassium channel (K_ATP_) currents in schwann cells and DRG plays a role in neuropathic pain development. Downregulation of potassium channels in DRG after nerve injury is also reported^13^. Further, the analgesic effect of potassium channel openers has been demonstrated in experimentally-induced neuropathic pain^14^.

Mitogen-activated protein kinases (MAPKs), a key group of serine-threonine kinases, play a crucial role in neuropathic pain signal transduction. Elevated expression of all MAPK members in DRG tissues has been observed in neuropathic pain models, whereas MAPK inhibition alleviate pain characteristics^15^. Among these, extracellular signal-regulated kinase 1/2 (ERK) is notably activated in neurons, astrocytes, microglia, and injured DRG neurons post-nerve injury, and its inhibition has been shown to relieve neuropathic pain in animal models^16–19^.

Nicorandil is a well-known K_ATP_ channel opener that is approved for treatment of angina pectoris^20^. Vasodilator action of nicorandil is mediated through its NO donor activity along with K_ATP_ channel opening activity^21^. We, among others, have demonstrated the antinociceptive and anti-inflammatory potential of nicorandil against different models of experimental pain^21–25^. Interestingly, our recent report^22^ showed that nicorandil antinociception is partially mediated via TRPV1/ opioidergic signaling. Yet, the underlying downstream signals of nicorandil mediated antinociception are still not fully elucidated.

An interaction between MAPKs, neuroinflammation, and oxidative stress is well-established. Activated members of MAPKs can trigger intracellular pathways in glial cells leading to the release of glial mediators including proinflammatory cytokines and ROS which contribute to pain potentiation through peripheral and central sensitization^26^. Additionally, ROS can activate MAPKs members by inhibition of MAPK phosphatases or by oxidative modulation of MAPK proteins^9^. Therefore, the present study investigated the antinociceptive activity of nicorandil against neuropathic and inflammatory chronic pain and the implication of NO/cGMP in addition to ROS/IL6-TNF-α/MAPK_ERK1/2_ pathways in chronic constriction injury model of sciatic nerve (CCI) and formalin test. Pharmacological, biochemical, histological and immunohistochemical studies were performed.

## Material and methods

### Drugs and reagents

Nicorandil (A gift from Adwia pharmaceuticals®, Egypt) was freshly prepared as suspension in 0.5% CMC in sterile saline. Nω-nitro-L-arginine methyl ester (L-NAME), L-arginine and naloxone were purchased from (Sigma Aldrich®). L-NAME was freshly prepared in saline (10 mg/ml). L-Arginine was freshly prepared in saline (200 mg/ml). Naloxone was freshly dissolved in saline (1 mg/ml). Methylene blue (MB) (from Merck) was freshly prepared in distilled water (5 mg/ml). Glibenclamide (gifted from Pharco Pharma for Pharmaceuticals, Egypt) was freshly prepared in DMSO: distilled water (3:1) in concentration 7.5 mg/ml. Sildenafil tablets (Viagra®, Pfizer) containing 100 mg were ground in mortar and dissolved in 100 ml distilled water and undissolved excipients were separated by filtration. Formalin 1% was prepared in saline immediately before experiments.

### Animals

In total, 128 male Wistar rats (180–230 g) were obtained from the animal house of faculty of pharmacy, Alexandria university. Rats were kept at room temperature and 40–60% humidity in a 12 h light/12 h dark cycle with free access to chow (19% protein, AL- Fajr feed Co., Egypt) and water. Effect of nicorandil on locomotor and exploratory activity were performed to rule out motor impairment or sedative effects that could falsely interfere with evaluating the antinociceptive potential of nicorandil. Then rats were randomly divided into two series including CCI and formalin test. The study was performed according to the institutional recommendations for animal care and use committee of the Faculty of Pharmacy, Alexandria University (Approval No. AU06201958150).

### Open field test

Open field test was conducted in a transparent acrylic box 46 × 46 × 23 cm. After a 10 min habituation period, rats were gently placed in the central zone of the transparent box and videotaped for 10 min. The apparatus was cleaned with 70% alcohol before each experiment. Total distance travelled, average speed, number of entries to central and peripheral zones and time spent in the central and peripheral zones were automatically analyzed using ANY-maze software (Stoelting, USA). The software divided the entire floor into sixteen squares (11.5 × 11.5 cm). The central zone consisted of the four central squares, while the remaining squares composed the peripheral zone. The number of rearing and grooming time were also measured. The experiment was conducted in the absence of the experimenter in the test room^27,28^.

### Chronic constriction injury (CCI) of sciatic nerve

First, thiopental sodium (50 mg/kg, I.P) was administered to rats for anesthesia. Then, blunt dissection through biceps femoris muscle was performed at the middle of the thigh to expose the sciatic nerve. Approximately 7 mm of the sciatic nerve was separated from the adhering tissue. Proximal to sciatic nerve trifurcation, four silk ligatures (4/0) were tied loosely around the sciatic nerve with 1 mm apart from each other. In sham rats, similar surgical procedures were performed without sciatic nerve ligation^29^. Nicorandil (150 mg/kg, twice, 2 h apart, PO) was administered on day 14 post surgery and mechanical allodynia was performed by von Frey test at 1, 3, 5 and 7 h after nicorandil first dose^25^. Cold allodynia was tested at 1.5, 3.5, 5.5 and 7.5 h after nicorandil first dose.

#### Assessment of mechanical allodynia in CCI rats

After habituation of rats in plastic cages located on a metal wire mesh floor (1 h/day for two consecutive days), paw withdrawal threshold was measured as the average of three measurements with 3 to 5 min interval between applications using electronic von Frey apparatus (panlab, Harvard, Spain). Paw withdrawal threshold was assessed before surgery (basal) and on alternate days after surgery to day 14^25,29^.

#### Assessment of cold allodynia in CCI rats

Acetone test was performed to assess cold allodynia in rats. Ten min after von Frey experiment, 50 µl of acetone was applied to mid-plantar skin of rat’s paw leading to cooling of the skin to non-noxious temperature (15–21 °C)^30^. Rats were observed for 1 min and scored according to the following scoring system: 0 for no response; 1 for mild paw withdrawal; 2 for prolonged flicking of hind paw or stomping; 3 for repeated flicking with licking hind paw. The cumulative score was measured for three trials with 2 min interval between successive applications^31^.

### Formalin test

After thirty min habituation in transparent acrylic box 46 × 46 × 23 cm, rats were injected with formalin solution (50 µl, 1%) in the plantar surface of the right hind paw (ipl)^32^. The time spent licking the paw and number of flinches were measured during two phases: phase 1 (0–10) and phase 2 (10–60 min)^33,34^. Nicorandil (150 mg/kg, twice, 2 h apart, PO) was administered 30 min prior to formalin injection.

### Blood samples collection

Rats were anesthetized using thiopental sodium (50 mg/kg, I.P) and blood was withdrawn from the retro-orbital plexus using glass capillary tubes^35^. Collected blood was allowed to clot at room temperature for 15 min, then centrifuged at 5000 rpm for 10 min. The resultant supernatant layer (serum) was aspirated, transferred into Eppendorf tubes, and stored at -80 °C till the time of analysis.

### Oxidative stress parameters

Oxidative stress was evaluated in serum of CCI rats by measuring malondialdehyde level (MDA) as thiobarbituric acid reactive substance. Samples were heated with thiobarbituric acid at acidic pH and the absorbance was measured for resulting color at 532 nm using spectrophotometer^36^.

### Serum analysis of inflammatory parameters by ELISA

Serum cyclooxygenase 2 (COX-2), tumor necrosis factor alpha (TNF-α) and interleukin 6 (IL-6) levels were measured using sandwich ELISA kits (CUSABIO, Novus Biologicals). The analysis was performed according to the manufacturer’s instruction.

### MAPK_ERK1/2_ and TRPV1 expression levels by immunohistochemistry

MAPK_ERK1/2_ and TRPV1 protein expression levels were evaluated in DRG immunohistochemically. After isolation of DRG tissues, tissues were fixed in 10% formaldehyde and then embedded in paraffin blocks. Four μm thick sections of DRG tissues were cut and placed on positively charged adhesive glass slides (Thermo Scientific, Berlin, Germany). Then, deparaffinization in xylene and rehydration in declining ethanol concentrations (100, 95 and 70%) were performed. For heat-induced epitope retrieval, slides were immersed in retrieval citrate buffer and placed in a microwave at power 100 for 1 min and then for 9 min at a power 30. Application of 0.3% hydrogen peroxide was performed for 10 min to block endogenous peroxidases. Then, primary polyclonal antibodies (rabbit anti-rat MAPK_ERK1/2_ and rabbit anti-rat TRPV1, 1:200, Sigma Aldrich company USA, Thermo Scientific®, Berlin, Germany, respectively) were applied to the slides which then incubated at 4 °C overnight. The secondary anti-rabbit antibody conjugated with horseradish peroxidase was applied to each section for 30 min. For protein visualization, the chromogen 3,3′-diaminobenzidine was added to each section as instructed by the manufacturer. Slides were immersed in hematoxylin for counterstaining and then in ascending concentrations of alcohol and xylene for dehydration. Images of DRG sections were taken by OptikamB9 digital camera (Optika Microscopes, Italy). Fiji Image J Software Version 1.51n (National Institutes of Health, Bethesda, Maryland, USA) was used to calculate the percentage of chromogen positive stained area in DRG.

#### Histopathological examination

After isolation of DRG (L4-L5) and sciatic nerve tissues (n = 5), tissues were fixed in 10% formalin and embedded in paraffin blocks. Then, 5 μm thick sections were cut and deparaffinized in xylene. Staining with hematoxylin and eosin (H&E) was performed. Evaluation of the stained sections were blindly performed using light microscope (Leica, DM500) and images were taken using a digital camera (EC3, Leica, Germany) at a magnification power of × 400^37^.

## Experimental groups and protocol

### Open field test

The effect of nicorandil (150 mg/kg, twice, 2 h interval, PO) on locomotor activity was conducted 7 h after first dose of nicorandil. Rats were randomly divided into two groups: 1) CMC, 2) nicorandil (150 mg/kg, twice, 2 h apart, PO).

#### Nociceptive tests

The effect of nicorandil (150 mg/kg, twice, 2 h interval, PO) was examined alone and in presence of L-arginine, methylene blue, sildenafil, L-NAME and glibenclamide in CCI and formalin nociceptive models.

*CCI model* Rats were randomly divided into sham group and seven CCI groups allocated to receive one of the following regimens: (1) CMC, (2) nicorandil (150 mg/kg, twice, 2 h apart, PO), (3) nicorandil + sildenafil (2.5 mg/kg, twice, 2 h apart, I.P), (4) nicorandil + L-NAME, (10 mg/kg, twice, 2 h apart, I.P), (5) nicorandil + L-arginine (500 mg/kg, I.P), (6) nicorandil + MB (10 mg/kg, I.P) and (7) nicorandil + glibenclamide (5 mg/kg, I.P).

Nicorandil (150 mg/kg, twice, 2h apart, PO) was administered on day 14 after surgery. L-NAME and sildenafil were administered 30 min before each dose of nicorandil. Glibenclamide, L-arginine and methylene blue were administered 30 min before the first dose of nicorandil. The above-mentioned pharmacological modulators were also investigated alone in CCI rats. At the end of the experiment, rats were anesthetized with thiopental (50 mg/kg, I.P), blood was withdrawn then rats were sacrificed using an overdose of thiopental (100 mg/kg, I.P), and finally ipsilateral sciatic nerves and L4-L5 DRG were isolated.

*Formalin test* Rats were randomly divided into eight groups (n = 5–8) one group received the vehicle and seven groups received formalin and allocated as follows: (1) control (CMC), (2) nicorandil (150 mg/kg, twice, 2 h apart, PO), (3) nicorandil + sildenafil (2.5 mg/kg, twice, 2 h apart, I.P), (4) nicorandil + L-NAME (10 mg/kg, twice, 2 h apart, I.P) (5) nicorandil + L-arginine (500 mg/kg, I.P), (6) nicorandil + MB (10 mg/kg, I.P) and (7) nicorandil + glibenclamide (5 mg/kg, I.P). The second dose of nicorandil was administered 30 min before formalin. The regimen of pharmacological modulators is mentioned in Section “CCI model”. The pharmacological modulators were investigated alone in formalin challenged rats.

**Fig. 1:**
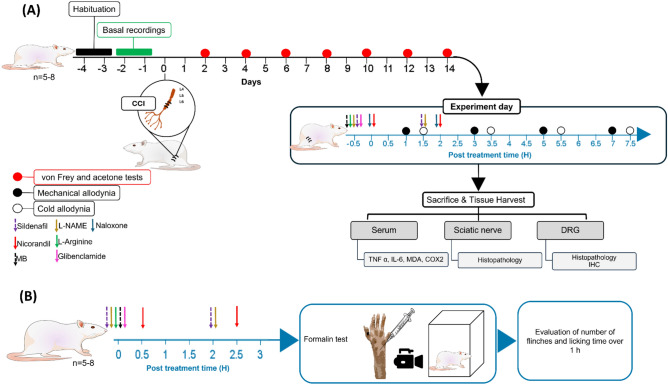
Experimental protocol and time schedule for drug administration in neuropathic pain model (**A**) and formalin model (**B**). CCI, chronic constriction injury of sciatic nerve; L-NAME, Nω-nitro-L-arginine methyl ester; MB, methylene blue; IHC, immunohistochemistry; DRG, dorsal root ganglia; TNF-α, tumor necrosis factor alpha; IL-6, interleukin 6; MDA, malondialdehyde and COX-2, cyclooxygenase-2.

The experimental protocol and time schedule for drug administration in neuropathic pain model (A) and formalin model (B) are illustrated in Fig. 1.

### Statistical analysis

The sample size for each experimental group was determined using G*Power software (version 3.1.9.7), with a significance level (α) of 0.05 and a statistical power of 80%. The effect size (Cohen’s d = 1.2) was derived from preliminary studies on nociceptive behavioral tests. Data are presented as mean ± standard error of the mean (SEM). For time-dependent measurements (mechanical and cold allodynia tests), statistical comparisons were performed using two-way ANOVA followed by Tukey’s post-hoc test. Results of open field test were analyzed using student unpaired t-test. For all other experiments, one-way ANOVA with Tukey’s post-hoc test was applied. A p-value < 0.05 was considered statistically significant. All statistical analyses were conducted using GraphPad Prism (version 8.0.2). Normality of data distribution was assessed using the Shapiro–Wilk test.

## Results

### Effect of nicorandil on locomotor activity in open field test

**Fig. 2:**
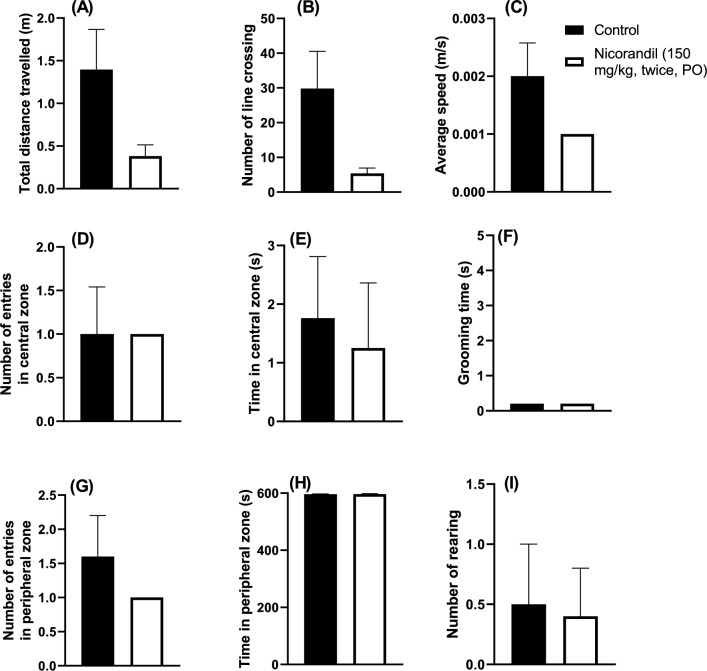
Effect of nicorandil (150 mg/kg, twice, 2 h apart, PO) on locomotor activity. Total distance travelled (**A**), Number of line crossing (**B**), average speed (**C**), number of entries in central zone (**D**), time spent in central zone (**E**), grooming time (**F**), number of entries in peripheral zone (**G**), time spent in peripheral zone (**H**) and number of rearing (**I**) assessed by open field test was evaluated 7 h after first dose of nicorandil. All results were expressed as mean ± SEM (n = 4–5). Results were analyzed by unpaired t-test.

The effect of nicorandil (150 mg/kg, twice, 2 h interval, PO) on locomotor activity was conducted in the open field test 7 h after first dose of nicorandil. As shown in Fig. 2, distance travelled, number of line crossing and average speed were insignificantly lowered in nicorandil treated rats compared to control rats (P > 0.05). Nicorandil did not alter other measured parameters including number of entries to the central and peripheral zones, time spent in the central and peripheral zones, number of rearing and grooming time (P > 0.05).

### Effect of pharmacological modulators of NO/cGMP/K_ATP_ signaling pathway on nicorandil mediated antinociceptive effect on mechanical allodynia induced by CCI

**Fig. 3:**
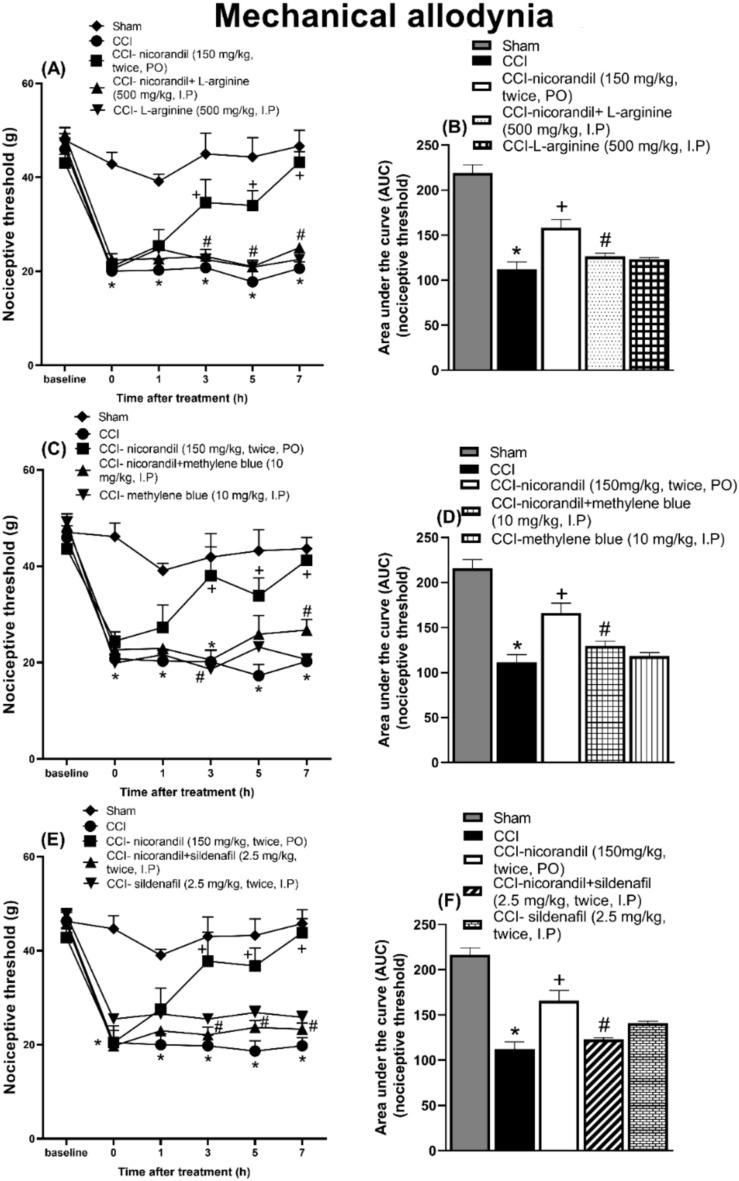
Effect of nicorandil (150 mg/kg, twice, 2 h apart, PO) on mechanical allodynia and the corresponding AUC induced by chronic constriction injury of sciatic nerve (CCI) in rats treated with L-arginine (A-B) (500 mg/kg, I.P, 30 min before first dose of nicorandil), methylene blue (C-D) (10 mg/kg, I.P, 30 min before first dose of nicorandil) and sildenafil (E–F) (2.5 mg/kg, I.P, 30 min before each dose of nicorandil). Mechanical nociceptive threshold assessed by von Frey test was evaluated at 1, 3, 5 and 7 h after first dose of nicorandil given on day 14 after surgery. All results were expressed as mean ± SEM (n = 5–8). Results were analyzed by two-way ANOVA in figures (A-C-E) and one-way ANOVA for figures (B-D-F) followed by Tukey’s post hoc test* P < 0.05 VS sham, + P < 0.05 VS CCI, # P < 0.05 VS CCI- nicorandil.

**Fig. 4:**
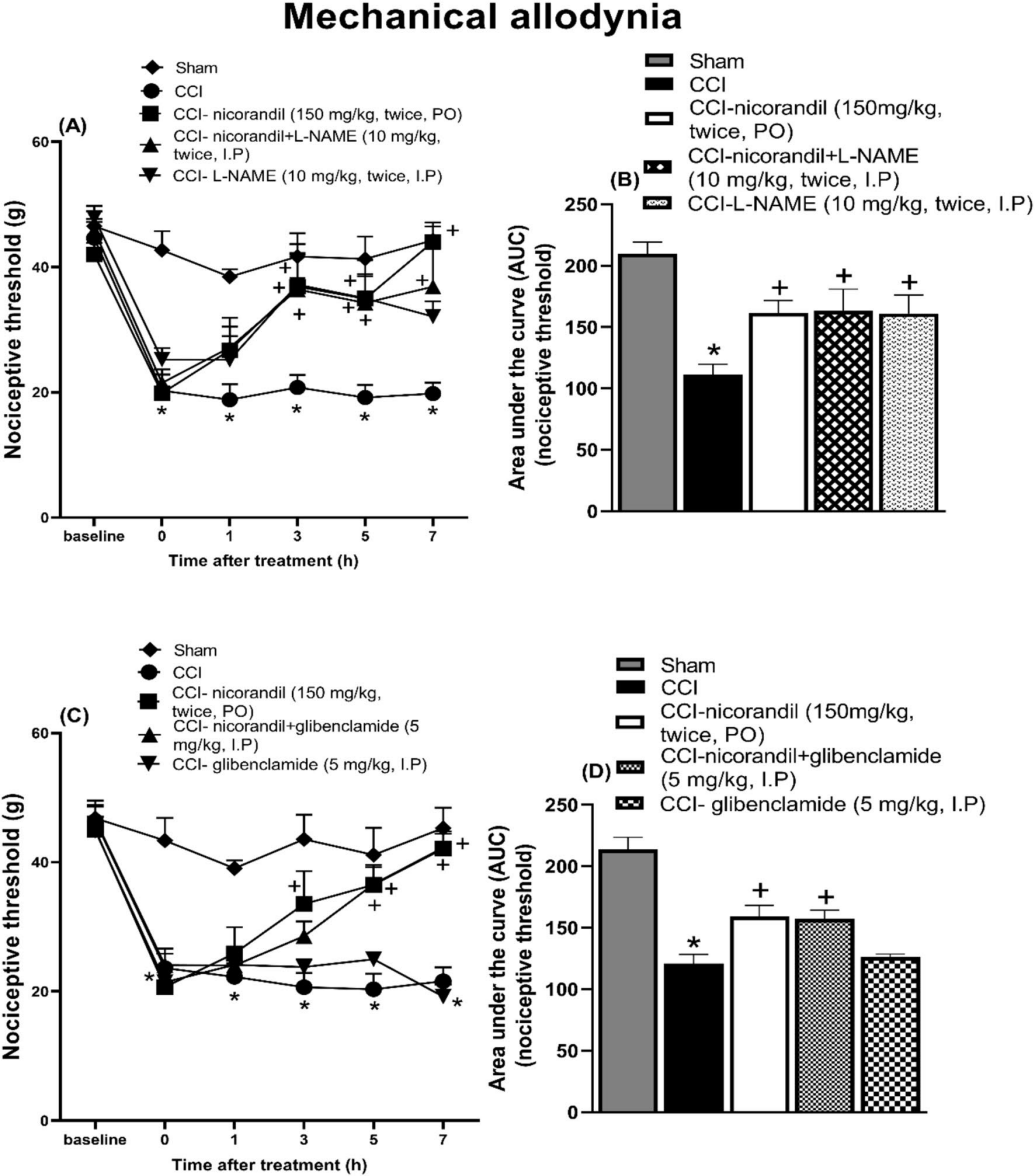
Effect of nicorandil (150 mg/kg, twice, 2 h apart, PO) on mechanical allodynia and the corresponding AUC induced by chronic constriction injury of sciatic nerve (CCI) in rats treated with L-NAME (**A**, **B**) (10 mg/kg, I.P, 30 min before each dose of nicorandil) and glibenclamide (**C**, **D**) (5 mg/kg, I.P, 30 min before first dose of nicorandil). Mechanical nociceptive threshold assessed by von Frey test was evaluated at 1, 3, 5 and 7 h after first dose of nicorandil given on day 14 after surgery. All results were expressed as mean ± SEM (n = 5–8). Results were analyzed by two-way ANOVA in figures (**A**–**C**) and one-way ANOVA for figures (**B**–**D**) followed by Tukey’s post hoc test* P < 0.05 VS sham, + P < 0.05 VS CCI.

Nicorandil (150 mg/kg, twice, 2 h apart, PO) significantly attenuated the mechanical allodynia observed at 3, 5 and 7 h intervals post administration of first dose of nicorandil (P < 0.05). The effect of nicorandil on mechanical allodynia was significantly abolished by prior administration of L-arginine (500 mg/kg, I.P) (P < 0.05) (Fig. 3A). CCI significantly reduced the calculated AUC of nociceptive threshold compared to sham rats (P < 0.05). Nicorandil partially reversed these falls in AUC of nociceptive threshold noted in CCI rats. Nicorandil’s effects were attenuated by prior administration of L-arginine (500 mg/kg, I.P) (P < 0.05) (Fig. 3B). Similarly, the antinociceptive effect of nicorandil on nociceptive threshold and the calculated AUC, was abolished by prior administration of methylene blue (10 mg/kg, I.P) (Fig. 3C, D) and sildenafil (2.5 mg/kg, twice, I.P) (P < 0.05) (Fig. 3E, F). L-NAME (10 mg/kg, twice, I.P) alone attenuated the mechanical allodynia at 3 and 5 h (P < 0.05). L-NAME (10 mg/kg, twice, I.P) did not alter nicorandil antinociception (P > 0.05) (Fig. 4A). Sole and combined administration of L-NAME (10 mg/kg, twice, I.P) with nicorandil significantly increased the calculated AUC of nociceptive threshold (P < 0.05) (Fig. 4B). Glibenclamide (5 mg/kg, I.P) did not alter the effect of nicorandil on the temporal course and the calculated AUC of the nociceptive threshold (P > 0.05) (Fig. 4C, D).

### Effect of pharmacological modulators of NO/cGMP/K_ATP_ signaling pathway on nicorandil mediated antinociceptive effect on cold allodynia induced by acetone in CCI rats

**Fig. 5:**
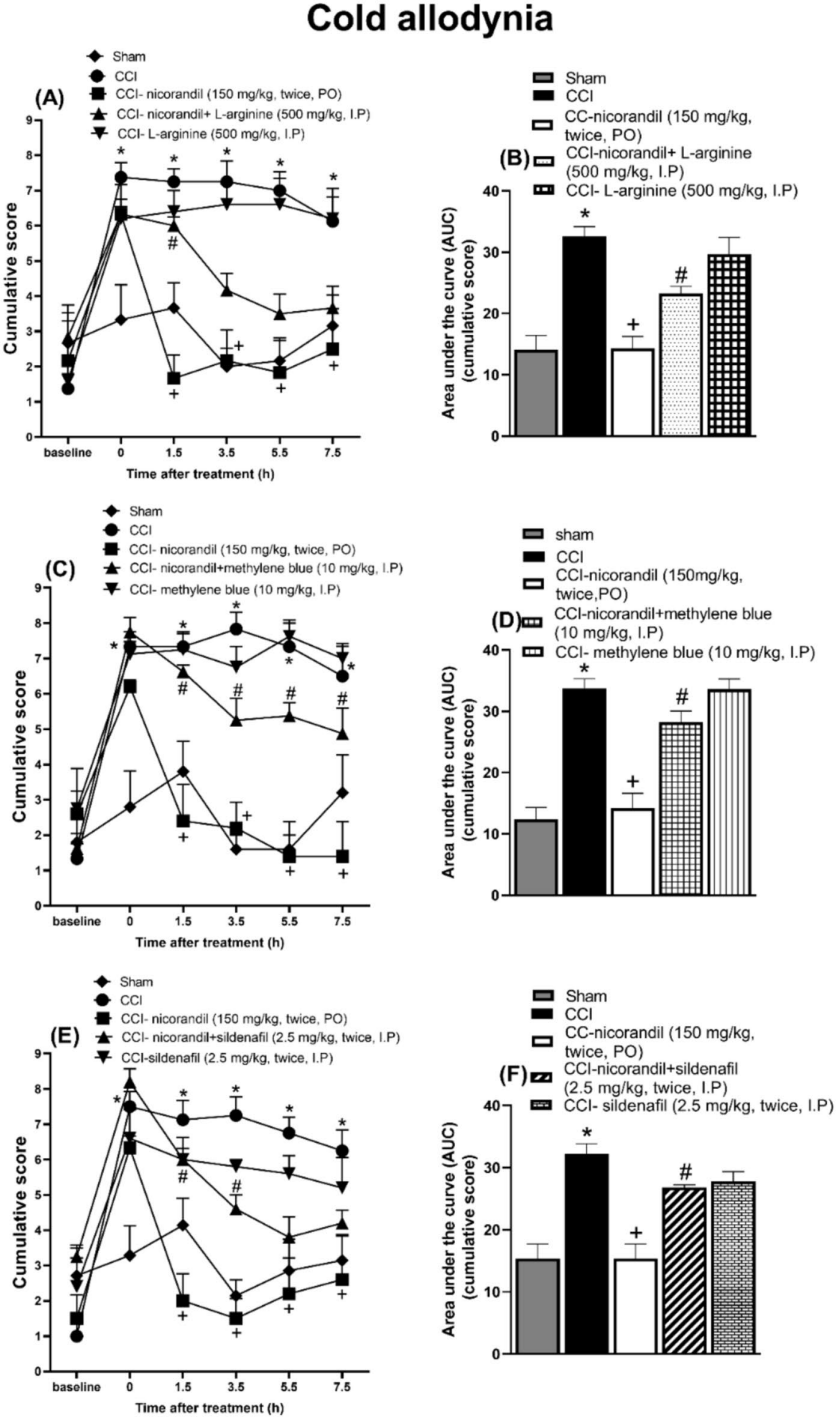
Effect of nicorandil (150 mg/kg, twice, 2 h apart, PO) on cold allodynia and the corresponding AUC induced by acetone after chronic constriction injury in rats (CCI) treated with L-arginine (A, B) (500 mg/kg, I.P, 30 min before first dose of nicorandil), methylene blue (C, D) (10 mg/kg, I.P, 30 min before first dose of nicorandil) and sildenafil (E, F) (2.5 mg/kg, I.P, 30 min before each dose of nicorandil). Cumulative score assessed by acetone test was evaluated at 1.5, 3.5, 5.5 and 7.5 h after first dose of nicorandil given on day 14 after surgery. All results were expressed as mean ± SEM (n = 5–8). Results were analyzed by two-way ANOVA in figures (A, C, E) and one-way ANOVA in figures (B, D, F) followed by Tukey’s post hoc test* P < 0.05 VS sham, + P < 0.05 VS CCI, # P < 0.05 VS CCI- nicorandil.

**Fig. 6:**
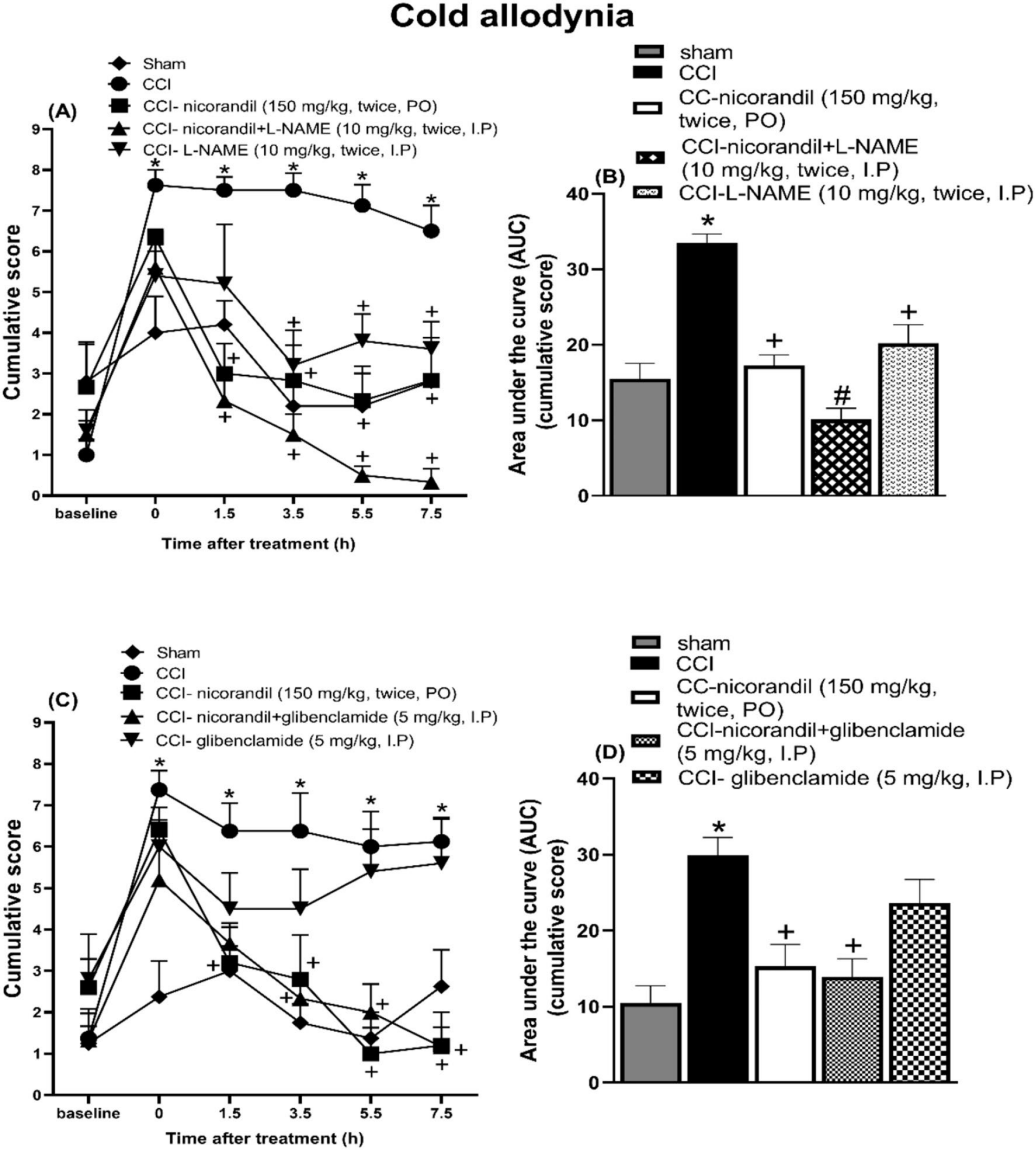
Effect of nicorandil (150 mg/kg, twice, 2 h apart, PO) on cold allodynia and the corresponding AUC induced by acetone after chronic constriction injury in rats (CCI) treated with L-NAME (A, B) (10 mg/kg, I.P, 30 min before each dose of nicorandil) and glibenclamide (C, D) (5 mg/kg, I.P, 30 min before first dose of nicorandil). Cumulative score assessed by acetone test was evaluated at 1.5, 3.5, 5.5 and 7.5 h after first dose of nicorandil given on day 14 after surgery. All results were expressed as mean ± SEM (n = 5–8). Results were analyzed by two-way ANOVA in figures (A, C) and one-way ANOVA in figures (B, D) followed by Tukey’s post hoc test* P < 0.05 VS sham, + P < 0.05 VS CCI, # P < 0.05 VS CCI- nicorandil.

Nicorandil administration (150 mg/kg, twice, 2 h interval, PO) significantly alleviated acetone cold allodynia at all tested time intervals (1.5, 3.5, 5.5 and 7.5 h after first nicorandil dose) manifested as significant decrease in the cumulative scores (P < 0.05). L-arginine (500 mg/kg, I.P) significantly attenuated the effect of nicorandil at 1.5 h post nicorandil injection (P < 0.05) (Fig. 5A). CCI significantly heightened the AUC of cumulative scores induced by acetone compared to sham rats (P < 0.05). Nicorandil significantly reduced the heightened AUC of acetone cumulative scores compared to CCI rats (P < 0.05). The effect of nicorandil was significantly abolished by prior administration of L-arginine (500 mg/kg, I.P) (P < 0.05) (Fig. 5B). Methylene blue (10 mg/kg, I.P) significantly abolished the effect of nicorandil at all tested time intervals and on the calculated AUC of cumulative scores (P < 0.05) (Fig. 5C, D). Sildenafil (2.5 mg/kg, twice, I.P) significantly abolished the effect of nicorandil at 1.5 and 3.5 h post nicorandil administration and on the calculated AUC of cumulative scores (P < 0.05) (Fig. 5E, F). L-NAME (10 mg/kg, twice, I.P) alone showed attenuation of cold allodynia at 3.5, 5.5 and 7.5 h. Combined with nicorandil, L-NAME attenuated the cold allodynia at all time intervals (P < 0.05) (Fig. 6A). L-NAME (10 mg/kg, twice, I.P) significantly potentiated nicorandil mediated reduction of the AUC of cumulative scores (P < 0.05) (Fig. 6B). Prior administration of glibenclamide (5 mg/kg, I.P) did not alter the effect of nicorandil on the temporal course and the calculated AUC of cumulative scores (P > 0.05) (Fig. 6C, D).

### Effect of pharmacological modulators of NO/cGMP/K_ATP_ signaling pathway on nicorandil mediated protective effect against formalin responses

**Fig. 7:**
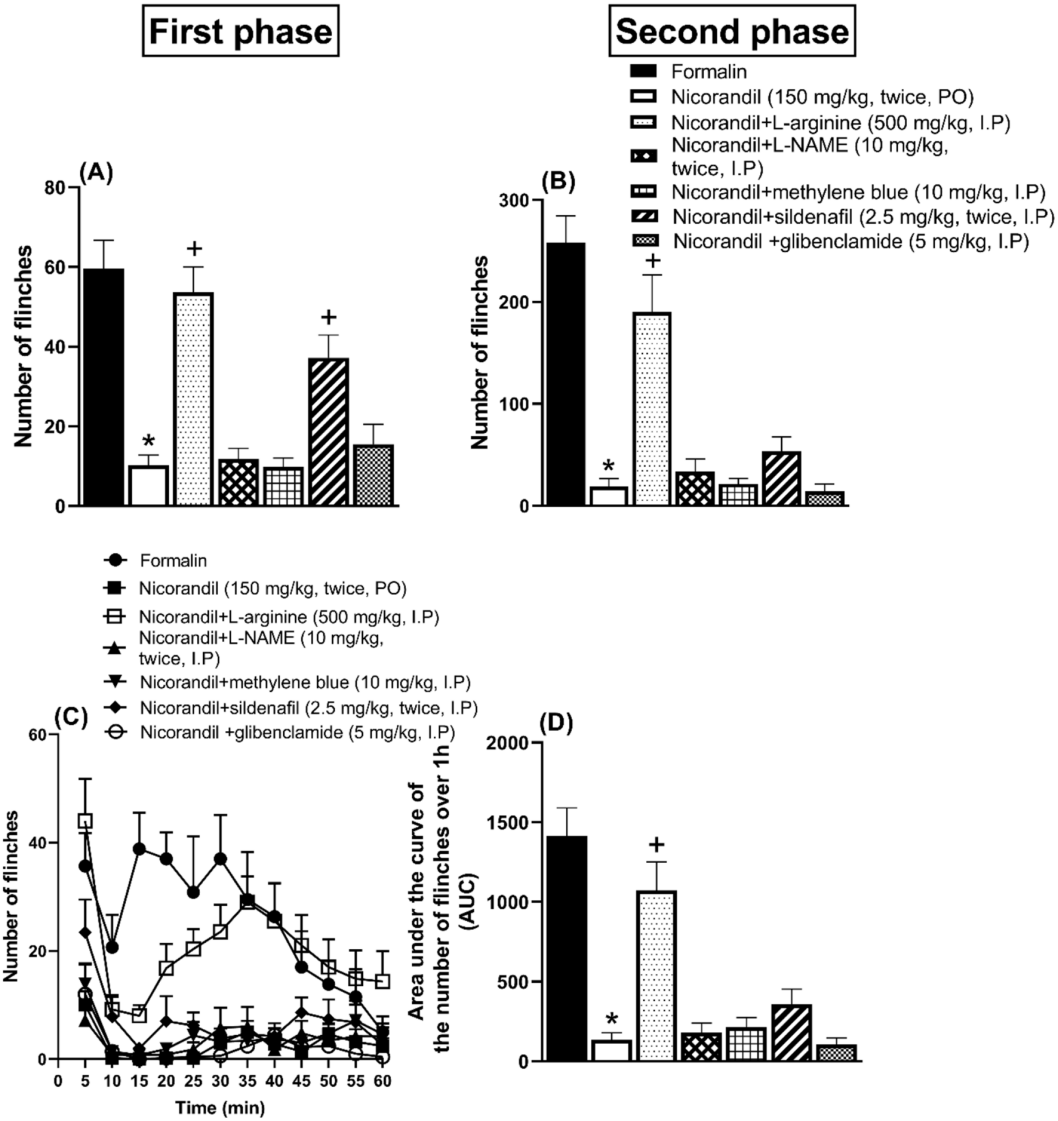
Effect of nicorandil (150 mg/kg, twice, 2 h apart, PO) on number of flinches of 1st, 2nd phases (**A**, **B**), number of flinches over 1 h (**C**) and total area under the curve for flinches (D) induced by formalin (50 µl, 1%) in presence of L-arginine (500 mg/kg, I.P, 30 min before first dose of nicorandil), L-NAME (10 mg/kg, I.P, 30 min before each dose of nicorandil), methylene blue (10 mg/kg, I.P, 30 min before first dose of nicorandil), sildenafil (2.5 mg/kg, I.P, 30 min before each dose of nicorandil) and glibenclamide (5 mg/kg, I.P, 30 min before first dose of nicorandil). All results were expressed as mean ± SEM (n = 5–8). Results were analyzed by one-way ANOVA followed by Tukey’s post hoc test *P < 0.05 VS formalin, + P < 0.05 VS nicorandil.

As depicted in Fig. 7, intraplantar administration of formalin (50 µl, 1%) caused licking and flinching responses of the injected hind paw. Prior administration of nicorandil (150 mg/kg, twice, 2 h interval, PO) significantly attenuated the licking time and number of flinches of the 1st and 2nd phases of formalin test, respectively, and reduced the AUC of total number of flinches (P < 0.05). Prior administration of L-arginine (500 mg/kg, I.P), L-NAME (10 mg/kg, twice, I.P), methylene blue (10 mg/kg, I.P), sildenafil (2.5 mg/kg, twice, I.P) and glibenclamide (5 mg/kg, I.P) did not change the effect of nicorandil on licking time in either 1st or 2nd phases of formalin test (Data not shown). L-arginine (500 mg/kg, I.P) significantly reversed the effect of nicorandil on flinches response of the 1st and 2nd phases of formalin test and increased the AUC of total number of flinches (P < 0.05) (Fig. 7A, B, D). Prior administration of sildenafil (2.5 mg/kg, twice, I.P) significantly blocked the effect of nicorandil during 1st phase flinches (P < 0.05) (Fig. 7A). Prior administration of L-NAME (10 mg/kg, twice, I.P), methylene blue (10 mg/kg, I.P) or glibenclamide (5 mg/kg, I.P) did not alter the effects of nicorandil on flinches’ response during the 1st and 2nd phases of formalin test (Fig. 7A–D). The time course of number of flinches induced by formalin administration (50 µl, 1%) over 1h in nicorandil treated rats in absence and presence of the previously mentioned pharmacological modulators was illustrated in Fig. 7C.

### Effect of nicorandil on serum MDA and COX 2 levels of CCI rats

**Fig. 8:**
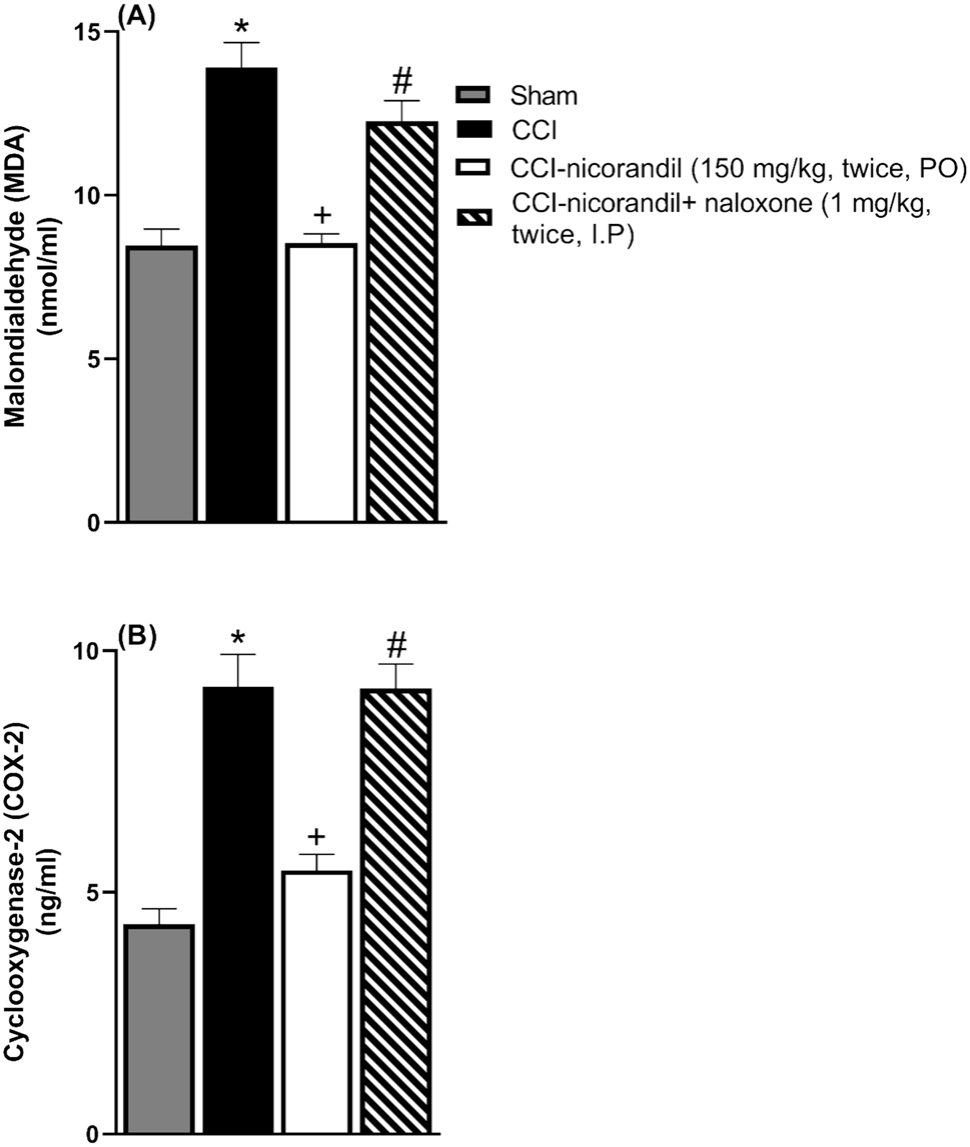
Effect of nicorandil (150 mg/kg, twice, 2 h apart, PO) in absence and presence of naloxone (1 mg/kg, twice, 2h apart, I.P) on malondialdehyde (MDA) (**A**) and cyclooxygenase 2 (COX-2) (**B**) serum levels in CCI rats. All results were expressed as mean ± SEM (n = 5). Results were analyzed by one-way ANOVA followed by Tukey’s post-hoc test. * P < 0.05 VS sham, + P < 0.05 VS CCI, # P < 0.05 VS CCI-nicorandil.

CCI significantly increased the level of MDA, a measure of oxidative stress, which was significantly reduced by the administration of nicorandil (150 mg/kg, twice, 2 h apart, PO) (P < 0.05) (Fig. 8A). CCI caused an increase in the serum COX 2 levels compared with sham rats (P < 0.05) (Fig. 8B). Nicorandil significantly reduced the heightened levels of serum COX 2 noted in CCI rats (P < 0.05). Naloxone (1 mg/kg, twice, 2h apart, I.P) pre-treatment abolished nicorandil-induced fall of serum MDA and COX 2 levels (P < 0.05) (Fig. 8A, B).

### Effect of nicorandil on serum levels of inflammatory cytokines in CCI rats

**Fig. 9:**
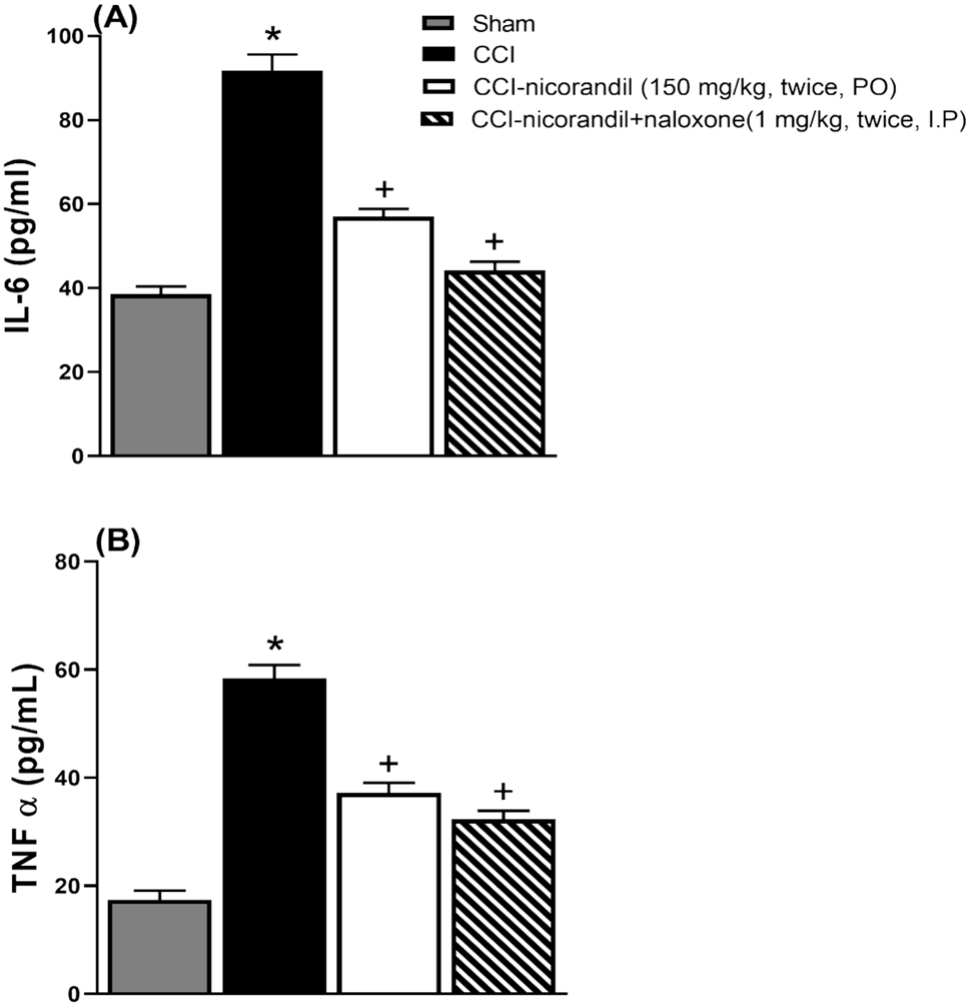
Effect of nicorandil (150 mg/kg, twice, 2 h apart, PO) in absence and presence of naloxone (1 mg/kg, twice, 2 h apart, I.P) on interleukin-6 (IL-6) (**A**) and tumor necrosis factor alpha (TNF-α) (**B**) serum levels in CCI rats. All results were expressed as mean ± SEM (n = 5). Results were analyzed by one-way ANOVA followed by Tukey’s post-hoc test. * P < 0.05 VS sham, + P < 0.05 VS CCI.

CCI caused a significant elevation in serum IL-6 levels compared to sham rats (P < 0.05). Nicorandil administration (150 mg/kg, twice, 2 h interval, PO) significantly decreased the heightened levels of serum IL-6 with % inhibition 37.9% (P < 0.05) (Fig 9A). In addition, CCI showed an increase in serum levels of TNF-α which was significantly attenuated by administration of nicorandil with % inhibition 36.3% (P < 0.05) (Fig 9B). Prior administration of naloxone (1 mg/kg, twice, 2 h apart, I.P) did not abolish the effect of nicorandil on TNF-α and IL-6 serum levels (P > 0.05) (Fig 9A, B).

### Effect of nicorandil on MAPK_ERK1/2_ expression levels in DRG of CCI rats

**Fig. 10:**
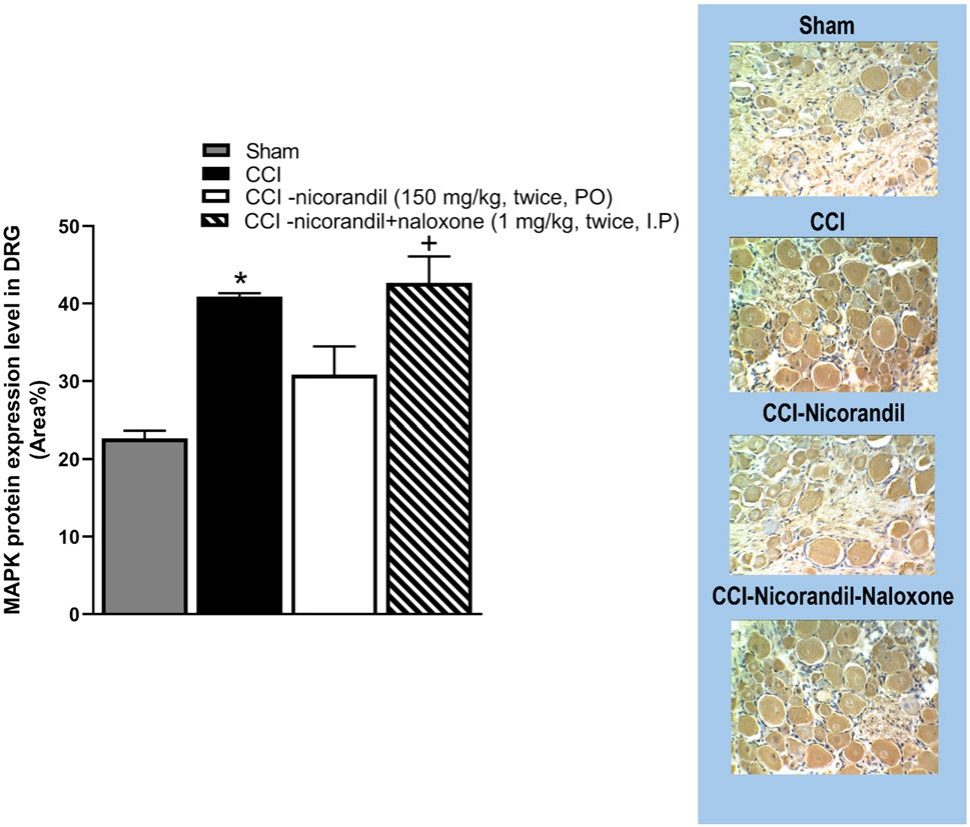
Effect of nicorandil (150 mg/kg, twice, 2h apart, PO) on immunohistochemical protein expression of MAPK_ERK1/2_ in DRG of CCI rats treated with naloxone (1 mg/kg, twice, 2h apart, I.P). Representative images for immuno-stained sections are also shown. All results were expressed as mean ± SEM (n = 5). Results were analyzed by one-way ANOVA followed by Tukey’s post hoc test. * P < 0.05 VS sham, + P < 0.05 VS CCI-nicorandil.

As illustrated in Fig. 10, immunohistochemical data showed significant increase in MAPK_ERK1/2_ protein expression levels in CCI rats compared to sham rats (P < 0.05). CCI failed to increase the MAPK_ERK1/2_ protein expression levels in nicorandil (150 mg/kg, twice, 2 h apart, PO) treated rats. In addition, prior administration of naloxone (1 mg/kg, twice, I.P) significantly reversed the nicorandil related fall in MAPK_ERK1/2_ levels in DRG tissues (P < 0.05).

### Effect of pharmacological modulators of NO/cGMP/K_ATP_ signaling pathway on nicorandil mediated changes in TRPV1 expression levels in DRG of CCI rats

**Fig. 11:**
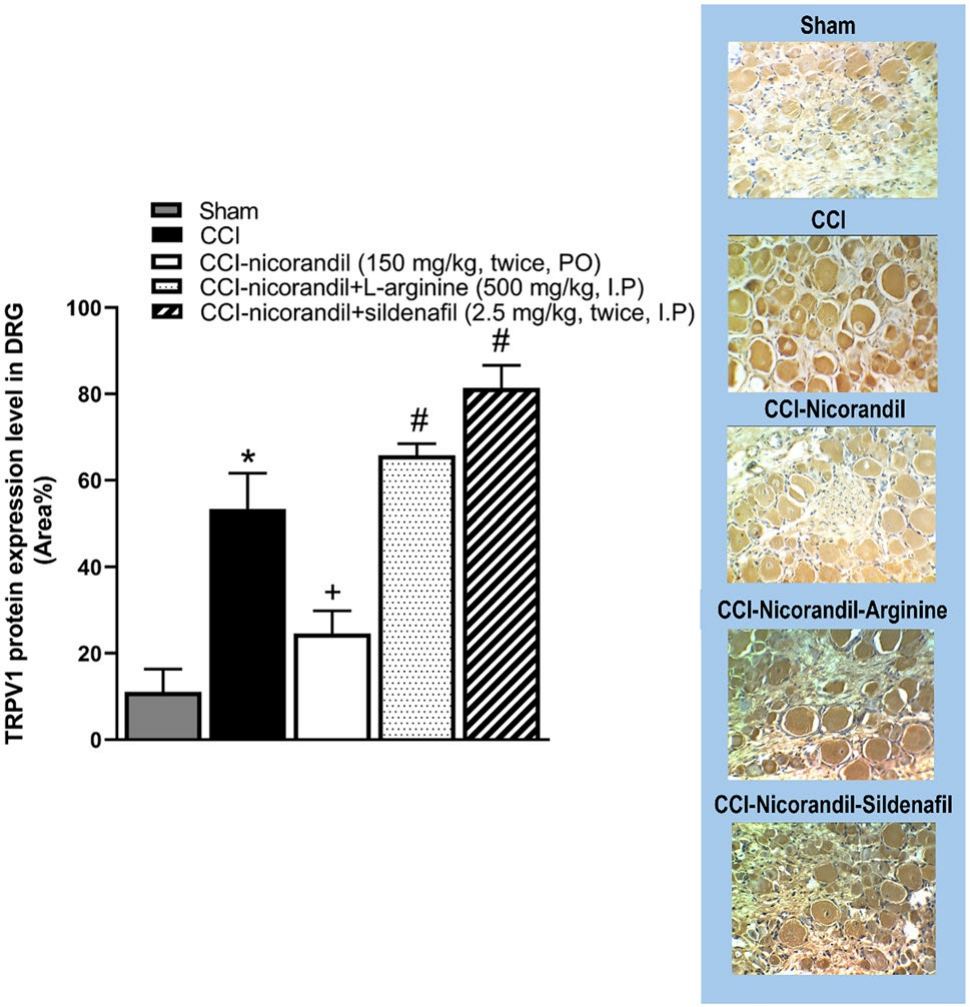
Effect of nicorandil (150 mg/kg, twice, 2h apart, PO) in presence of L-arginine (500 mg/kg, I.P) and sildenafil (2.5 mg/kg, twice, I.P) on immunohistochemical protein expression of TRPV1 channels in DRG of CCI rats. Representative images for immuno-stained sections are also shown. All results were expressed as mean ± SEM (n = 5). Results were analyzed by one-way ANOVA followed by Tukey’s post-hoc test. * P < 0.05 VS sham, + P < 0.05 VS CCI, #P < 0.05 VS CCI-nicorandil.

As shown in Fig. 11, immunohistochemical data showed that TRPV1 protein expression level was heightened in CCI rats compared to sham (P < 0.05). Interestingly, CCI failed to increase the expression of TRPV1 in nicorandil (150 mg/kg, twice, 2 h apart, PO) treated rats. However, L-arginine (500 mg/kg, I.P) and sildenafil (2.5 mg/kg, twice, I.P) reversed the nicorandil related falls in TRPV1 expression levels (P < 0.05).

### Effect of pharmacological modulators of NO/cGMP/K_ATP_ signaling pathway on nicorandil mediated histopathological changes in CCI rats

**Fig. 12:**
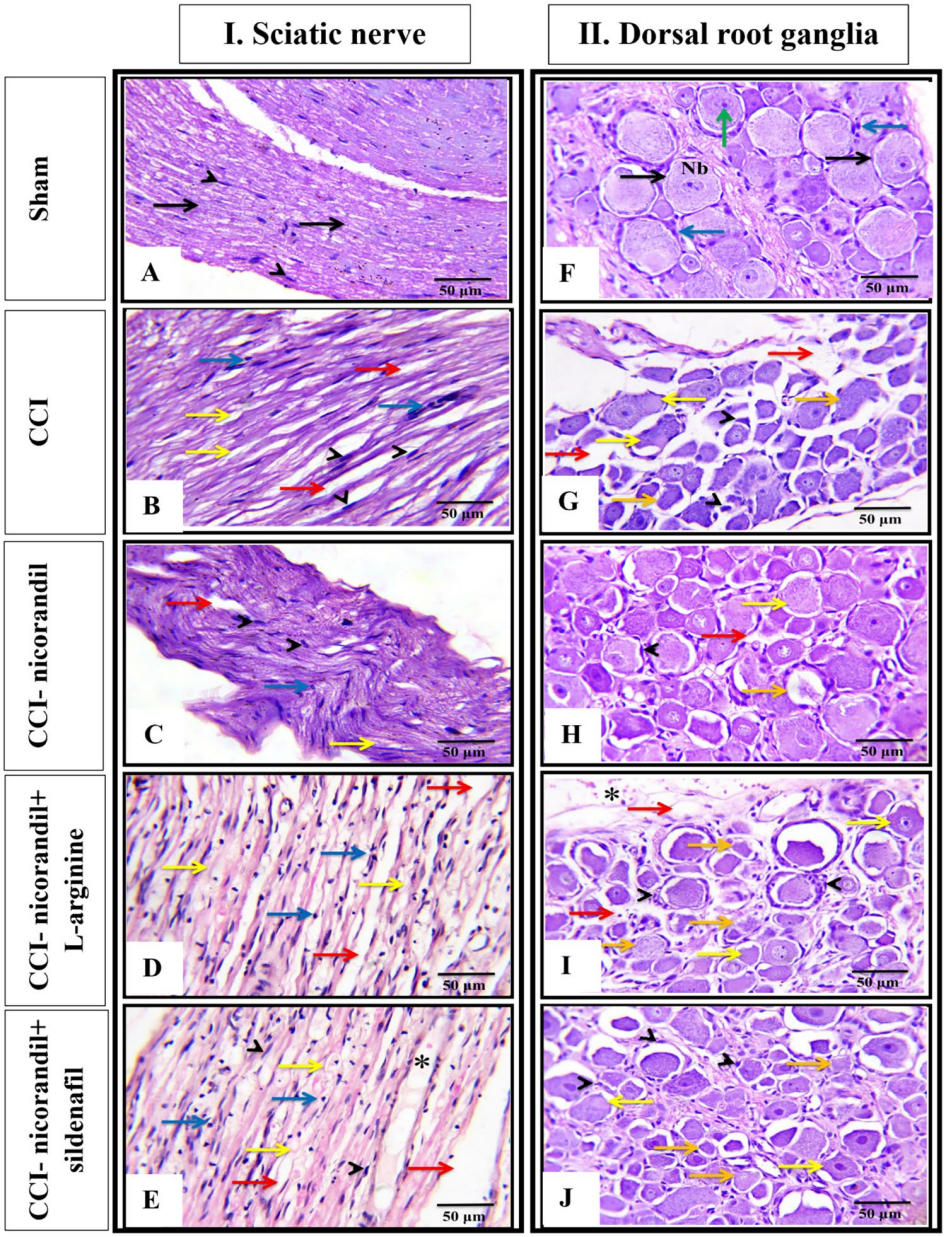
Effect of nicorandil (150 mg/kg, twice, 2 h apart, PO) on CCI induced histopathological alterations in longitudinal sections of sciatic nerve (**A**–**E**) and transverse section of dorsal root ganglia (**F**–**J**) of sham (**A**, **F**), CCI (**B**, **G**), nicorandil (150 mg/kg, twice, 2 h apart, PO) treated CCI rats (**C**, **H**), L-arginine (500 mg/kg, I.P) pretreated CCI rats (**D**, **I**) and sildenafil (2.5 mg/kg, twice, I.P) pretreated CCI rats (**E**, **J**), respectively. All images were captured under magnification power X400 (scale bar: 50 µm) (n = 5). Sciatic nerve: Normal nerve fibers (black arrow), myelin sheath degeneration (yellow arrow), areas of edema (red arrow), Schwann cells nuclei (arrow heads), mononuclear cells infiltration (blue arrow), increase the capillary size (*). Dorsal root ganglia: Normal nerve cells (black arrow), Nissl body in the cytoplasm of nerve cells (Nb), Satellite cells (blue arrow), Nucleus with prominent nucleolus (green arrow), Degenerated neurons with absence of Nissl bodies (yellow arrow), Areas of edema (red arrow), Hypertrophy of satellite cells (arrow heads), The neuronal cells showed loss of the nuclei and showed a ghost cell-like morphology (orange arrow).

Microscopic examination of longitudinal sections of sciatic nerve tissues is illustrated in Fig. 12 (panel Ⅰ A-E). Sciatic nerves of sham group (control) showed normal architecture of nerve fibers with normal myelin sheaths and schwann cells’ nuclei. In addition, there is no apparent inflammatory infiltrating cells (Fig. 12A). However, longitudinal sections of sciatic nerves of CCI group showed degenerated nerve fibers and myelin sheath as well as Wallerian degeneration of sciatic axon and infiltrating inflammatory cells. Increased number of fibroblasts and schwann cells and increased capillaries size with surrounding edema in CCI sciatic nerve were also observed (Fig. 12B). Interestingly, partial restoration of normal sciatic nerve structure was observed in nicorandil treated CCI group (Fig. 12C). Prior administration of L-arginine and sildenafil partially attenuated nicorandil effect as observed by increased mononuclear infiltrating cells, area of edema and myelin sheath degeneration (Fig. 12D, E).

Microscopic examination of transverse sections of DRG tissues is shown in Fig. 12 (panel Ⅱ F-J). DRG tissues of sham group showed normal architecture of the ganglion cells with abundant cytoplasm and Nissl bodies and normal satellite cells surrounding the ganglion cells (Fig. 12F). Conversely, DRG tissues of CCI rats showed nerve cell degeneration, disappearance of Nissl bodies and ghost like morphology (Fig. 12G). Surprisingly, DRG tissues of nicorandil treated group showed recovery of nerve cell structure and re-appearance of Nissl bodies and nuclei (Fig. 12H). Prior administration of L-arginine and sildenafil partially reversed the nicorandil beneficial effects observed as increase in degenerated neurons, ghost like morphology cells and hypertrophy of satellite cells (Fig. 12I, J).

## Discussion

The current study reports on the antinociceptive mechanisms of nicorandil in neuropathic and inflammatory pains induced by CCI and formalin, respectively, and the molecular bases of nicorandil antiallodynic effect. First, nicorandil induced antinociception was substantially attenuated following the exposure to a nitric oxide precursor and enhancer suggesting a prime functional role of NO/cGMP in mediating neuropathic pain. Second, blockade of ATP-dependent potassium channels does not seem to play a pivotal role in nicorandil antiallodynic response. Third, nicorandil reduced oxidative stress and cytokine production and attenuated the CCI upregulated protein expression of MAPK_ERK1/2_ in DRG associated with restored histological structural integrity of sciatic nerve and DRG.

The open field test for locomotor activity can recognize the confounding motor or sedative effects that may falsely interpreted as antinociception^38^. Current findings showed that nicorandil did not influence locomotor and exploratory activity. Therefore, the antinociceptive activity of nicorandil is unlikely to be a result of motor impairment. Consistent with our findings, single^23^ and multiple doses^25^ of nicorandil did not alter the motor function assessed at similar time point recorded in our study. Furthermore, chronic administration of nicorandil did not influence the locomotor and exploratory activity assessed in a model of traumatic brain injury by performing open field test^39^.

The antinociceptive effect of nicorandil was attenuated by NO/cGMP modulators, as evidenced by prior administration of the NO precursor, arginine and the phosphodiesterase inhibitor, sildenafil. NO has a crucial complex role in nociceptive transmission and is reported to have a dual effect on nociception^40^. During central sensitization, activation of NMDA receptors results in production of NO which activates neighboring neurons and astrocytes leading to hyperalgesia and allodynia^41,42^. The nociceptive effect of NO is mediated via production of cGMP which has several targets including ion channels, PKG and phosphodiesterase^42,43^. Additionally, peripheral NO nociception can be mediated by activation of cyclooxygenase enzyme and production of prostaglandins^40^. Confirming this notion, the current results showed that nicorandil inhibited COX2 activity which contributed to its analgesic action. The NO precursor, L-arginine is reported to increase hyperalgesia^44,45^. In addition, elevation of nitrite and nitrate levels in ligated sciatic nerve confirmed the role of NO in CCI induced neuropathy^45^. That said, NO/cGMP pathway is activated in the spinal cord and DRG contributing to the incidence of neuropathic pain^11^.

The results of NOS inhibition are controversial. Contrary to the enforcing impact of NO/cGMP on pain perception, the present findings indicated that the NOS inhibitor L-NAME attenuated neuropathic pain and potentiated nicorandil’s analgesia in CCI neuropathic rats. Following nerve injury, NOS inhibitors are reported to attenuate the behavioral signs of neuropathic pain in neuropathic models indicating the importance of NO in maintenance of neuropathic pain^46,47^. On the other hand, the NOS inhibitor was reported to inhibit the beneficial effects of nicorandil^48–50^ without altering the antinociceptive effect of nicorandil/morphine combination in tail flick and formalin test in liver fibrotic rats^48^. Conjointly, the current data showed that NOS inhibition did not modify the antinociceptive effect of nicorandil on mechanical allodynia and formalin test but potentiated its effect on acetone cold allodynia.

Despite being odd, the NO donor nicorandil is suggested to mediate antinociception via reduction of NO rather than increasing it, that does pose an oxidative stress on multiple tissues including neurons that aggravate neuropathic pain. Negative feedback inhibition of NOS enzyme by excessive NO levels leads to enzyme inactivation^51,52^. Therefore, high NO concentration detected in the pathophysiology of neuropathic pain together with nicorandil NO donation may result in negative feedback inhibition of NOS enzymes. It is possible that an inhibitory action of nicorandil on NO production underlies its antinociceptive potential against neuropathic and inflammatory pains. Moreover, nNOS enzyme is one of the intracellular targets of TRPV1 leading to production of NO. The peripheral nociceptive activity of NO is mediated through TRPV1 and TRPA1 activation^53^. Collectively, the reported interaction between TRPV1 and NO pathways along with current findings regarding nicorandil modulation of TRPV1^22^, warrant further investigation. Nevertheless, the antinociceptive activity of moderate levels of NO cannot be ignored and is mediated via cGMP/PKG/K_ATP_ pathway which is involved in opioids and nonsteroidal anti-inflammatory drugs induced analgesia^40^.

Expectedly, the current data showed that sildenafil antagonized the antinociceptive effect of nicorandil in both CCI model and formalin test. cGMP/PKG pathway is critical for maintenance of DRG neuronal hyperactivity. Inhibition of cGMP/PKG pathway suppresses the hyperexcitation of DRG and relieves neuropathic pain^54^. Reduction of cGMP levels in neuronal cells is reported to alleviate neuropathic pain in spared injury model in rats^55^. NO/GC knockout mice showed diminished nociceptive response to neuropathic and inflammatory pain^56^. Intrathecal sildenafil is reported to induce hyperalgesia in CCI rats through elevation of cGMP levels and PKG activation^57^.

The comparable effects of NO enhancement and inhibition by sildenafil and methylene blue, respectively, seem quite contradictory since both agents reversed nicorandil mediated antinociception. The key point is that, the antinociceptive effect of nicorandil in CCI model was abrogated by methylene blue. The blocking effect may be due to the neurotoxicity of high concentrations of methylene blue that cause peripheral neuronal toxicity, neuronal loss and alteration of membrane electrical properties in ganglia neurons^58^. In addition, a single bolus IV dose of methylene blue in rats caused apoptotic effect after injection in the rat brain indicating its neurotoxicity in the CNS^59^. Nicorandil antinociception was abrogated by methylene blue in tail flick and formalin test in liver fibrotic rats and ODQ—a selective inhibitor of soluble guanylyl cyclase- in formalin test^23,48^. Another possible explanation of the inhibitory effect of methylene blue of nicorandil’s analgesia may be due to serotonergic interaction rather than guanylyl cyclase inhibition. Administration of methylene blue with serotonergic medications caused serotonin toxicity via inhibition of monoamine oxidase-A^60^. In support, nicorandil beneficial effect in paclitaxel induced neuropathic pain, was mediated via serotonergic pathway^25^. Reportedly, serotonin has both hyperalgesic and analgesic actions depending on the subtype of receptors and the location of serotonin action^61^.

It is important to comment on the failure of glibenclamide, the K_ATP_ blocker, to attenuate nicorandil’s antinociception. Glibenclamide did not reverse the antinociceptive effect of nicorandil in nociceptive threshold and cumulative scores assessed by von Frey and acetone test in CCI rats, respectively, or block the effect of nicorandil in formalin test. The release of NO and opening of K_ATP_ channels may contribute to the vasodilator activity of nicorandil^62–64^ and pain relief in patients with myocardial ischemia^20,65^. Activation of K_ATP_ channels causes hyperpolarization of nociceptive neurons, suppresses pain sensation^66^ and can reduce postoperative pain in rats^67,68^. Notably, nicorandil is the first clinical K_ATP_ opener, which has been reported to exhibit antinociception against inflammatory and nociceptive pain^23,24^. Nicorandil either tested alone^23–25,69^ or in combined therapy^48,70^, exhibited antinociception in different pain models. Nicorandil inhibited mechanical allodynia induced by paclitaxel by activating opioidergic but not K_ATP_ channels^25^. Glibenclamide administration did not reverse nicorandil’s analgesia in formalin test^23^ and tail flick test in liver fibrotic rats^48^. Therefore, based on the current findings and supported by previous reports, it is unlikely that K_ATP_ channels play a major role in the mechanism of action of nicorandil in treatment of neuropathic pain. Nevertheless, nicorandil antinociception against mechanical allodynia induced by other insults^69^ was blocked by prior administration of glibenclamide^71^.

Several mediators are involved in neuropathic pain such as production of NO, ROS, TNF-α, IL6 as well as activation of MAPK_ERK1/2_ and TRPV1 channels which interplay together to generate pain. At this point we aimed to investigate whether the inhibition of NO pathway by nicorandil could impact the intracellular downstream signals of MAPK and to clarify whether it is modified by oxidative stress and inflammation. For this purpose, levels of COX2, which initiates the formation of inflammatory and painful prostaglandins, MDA, a reliable marker of lipid peroxidation and oxidative stress^72^, and TNF-α/ IL-6, potent inflammatory cytokines in rats were estimated.

Herein, nicorandil decreased the levels of serum COX2 and MDA in CCI rats; an effect that was abrogated by naloxone. Oxidative stress is linked to initiation and maintenance of neuropathic pain^73^. CNS injury together with stimulation of NMDA receptors by high glutamate levels result in calcium ions influx producing excessive release of ROS^74,75^. Conversion of arachidonic acid to prostaglandins, is catalyzed by COX enzymes^76^. Nicorandil limits oxidative stress and decreases COX2 expression levels^77^. Nerve damage during neuropathic pain triggers oxidative stress by upgrading oxidant species such as MDA. Further, nicorandil reduced the heightened MDA levels in acetaminophen induced hepatic insult^78^. The current results, in accordance, confirmed the antioxidant profile of nicorandil as evidenced by the reduction of serum MDA level as previously reported^79–81^.

Oxidative stress and neuroinflammation are intricately linked to neuropathic pain^8^. Current findings revealed that levels of TNF-α and IL6 were substantially increased in CCI rats and attenuated by nicorandil treatment. Sensitization of nociceptors by inflammatory mediators such as cytokines can lead to persistent neuropathic pain^82^. Nicorandil limits the inflammatory cell infiltration^83,84^ and inhibits the release of proinflammatory cytokines such as TNF-α and IL1β^85^. ROS have multiple roles in neuropathic pain. First, excitation of nociceptive neurons via disruption of glutamate pathway and activation of NMDA receptors^9^. Second, activation of TRP channels^86^. Finally, activation of MAPK pathway contributing to pain sensitization after nerve damage^87^.

Predictably, there is a crosstalk between NO and other signaling pathways. Mutually, after nerve injury, all components of MAPK are activated that trigger the release of proinflammatory cytokines and production of ROS^9^ which activate glial and immune cells to maintain neuroinflammation^9^. Immunohistochemical analysis, while providing valuable spatial protein expression data, revealed that nicorandil blunted the CCI-induced elevation of MAPK-ERK1/2 phosphorylation and TRPV1 protein levels in DRG neurons. Consistently, nicorandil exhibited multiple protective effects via inhibition of MAPK activity. Nicorandil showed neuroprotective effect against autoimmune encephalomyelitis via downregulation of P38/JNK-MAPK protein expression and TNF-α and IL-6^88^. Additionally, nicorandil alleviated acute lung injury^85^ and apoptosis through MAPK signaling pathways in human pulmonary artery endothelial cell^89^. Moreover, nicorandil ameliorated doxorubicin-induced nephrotoxicity, reversed the heightened renal expression level of P38 MAPK^90,91^ and suppressed the activation of MAPK_ERK_ in rat pheochromocytoma PC12 cells^92^. Finally, nicorandil inhibited the phosphorylation of ERK in rat cardiac fibroblast cells^93^. Collectively, it seems clear that nicorandil attenuated the MAPK_ERK1/2_ protein expressions in DRG of CCI rats is linked to the antinociception in neuropathic pain.

Expression of TRPV1 channels is transcriptionally controlled by stimulation of MAPK pathway^94^. Upregulation of MAPK_ERK1/2_ and TRPV1 channels triggered neuropathic pain while their downregulation alleviated pain^18^. All these maneuvers were orchestrated by NO signaling. Therefore, it is plausible to suggest that NO increases cGMP and the downstream signals of nicorandil’s antinociception. Nicorandil mediated TRPV1 antagonistic activity is due to direct interaction with TRPV1 channels^22^ or through blunting inflammatory mediators and ROS activity and downregulation of MAPK_ERK1/2_. TNF-α is reported to induce elevation in TRPV1 expression in DRG neurons, which is mediated via ERK activation^95^. According to the current findings and others^18,96^, downregulation of both TRPV1 and MAPK_ERK1/2_ can alleviate neuropathic pain. MAPK is involved in many biological actions. Nerve injury of neurons and microglia activates MAPK_ERK_ phosphorylation which could contribute to iNOS induction and NO production^17^ which plays a crucial role in processing of neuropathic pain^97^. Furthermore, NO donors induce MAPK_ERK1/2_ and p38 MAPK phosphorylation in cardiomyocytes^98,99^. In the present study, nicorandil reduced MAPK_ERK1/2_ level in DRG and inhibited NO pathway leading to relieved neuropathic pain.

The crucial role of NO on nicorandil induced down regulation of TRPV1 receptors should be verified. NO was reported to stimulate TRP channels causing Ca^+2^ entry into cells via s-nitrosylation of the cysteine moiety of TRP channels^100^. TRPV1 can affect NO synthesis by regulating nNOS enzyme activity^101^. Previous studies have shown that systemic administration of capsaicin, a potent TRPV1 receptor antagonist, enhanced NO production in different brain regions in rats^102,103^ that was attenuated by selective nNOS inhibitors and potentiated by L-arginine^101^. In the current study, nicorandil’s downregulatory effect on TRPV1 channels in DRG was reversed by L-arginine and sildenafil confirming the importance of NO pathway in nicorandil TRPV1 downregulatory effect. Moreover, the central role played by NO extends beyond its antinociceptive benefits. Nicorandil exerted protective effect on histological insults induced by CCI in sciatic nerve and DRG as indicated by restored cellular integrity and retrieval of normal architecture of nerve fibers. Nicorandil, acting as a mitochondrial K_ATP_ channel opener and NO donor, may rapidly enhance microvascular perfusion and mitochondrial function, thereby mitigating early axonal swelling and fragmentation observed in our study. This interpretation is consistent with previous reports of nicorandil’s acute cytoprotective effects and provides a plausible explanation for the early morphological changes observed^71,104,105^. These beneficial effects were partially attenuated by concurrent administration of nitric oxide precursor and enhancer, L-arginine and sildenafil, respectively.

Notably, in addition to the involvement of opioid signaling in nicorandil antinociception^22^, it is imperative to reveal that naloxone reversed the other beneficial effects of nicorandil. The inhibition of opioid receptors recalled the deficits induced by CCI on ROS, MAPK_ERK1/2_ protein expression in addition to reappearance of histological insults.

### Study limitations and future perspectives

The present study, while demonstrating the acute antinociceptive and anti-inflammatory effects of nicorandil in experimental models, has certain limitations that should be acknowledged. First, the experimental design utilized an acute, interventional approach, assessing the antinociceptive effects of nicorandil within a short time window (7 h post-administration). While demonstrating a beneficial potential, it does not provide information on the long-term efficacy or tolerability of nicorandil under chronic pain conditions. Second, while the systemic (oral) route is the clinically adopted route of administration, it doesn’t indicate the primary site of action of nicorandil; peripheral/central actions. Third, the assessment of key molecular targets, namely MAPK-ERK1/2 and TRPV1, relied on immunohistochemistry. Although this method provided valuable protein expression data within the complex architecture of the DRG, it is considered semi-quantitative. More quantitative techniques such as Western blotting or analysis of mRNA levels via qRT-PCR will be considered in future studies to strengthen the molecular conclusions, particularly for detecting rapid transcriptional changes. Finally, the investigation focused primarily on peripheral mechanisms in the DRG and sciatic nerve; the potential role of spinal cord or supraspinal NO/cGMP and MAPK signaling in nicorandil’s antinociception remains to be explored. Future studies employing ex vivo electrophysiology on isolated DRG neurons or detailed molecular profiling (RNA-seq) of pain pathways would be invaluable to confirm and extend these mechanistic insights at a cellular level. Furthermore, time-course studies measuring both mRNA (by qRT-PCR) and protein levels of these targets would help delineate the sequence of molecular events.

Despite these limitations, the current findings robustly indicate that nicorandil, through its multifaceted action on NO signaling, oxidative stress, inflammation, and key pain-pathway proteins, represents a promising candidate warranting further investigation for the management of neuropathic pain.

**Fig. 13:**
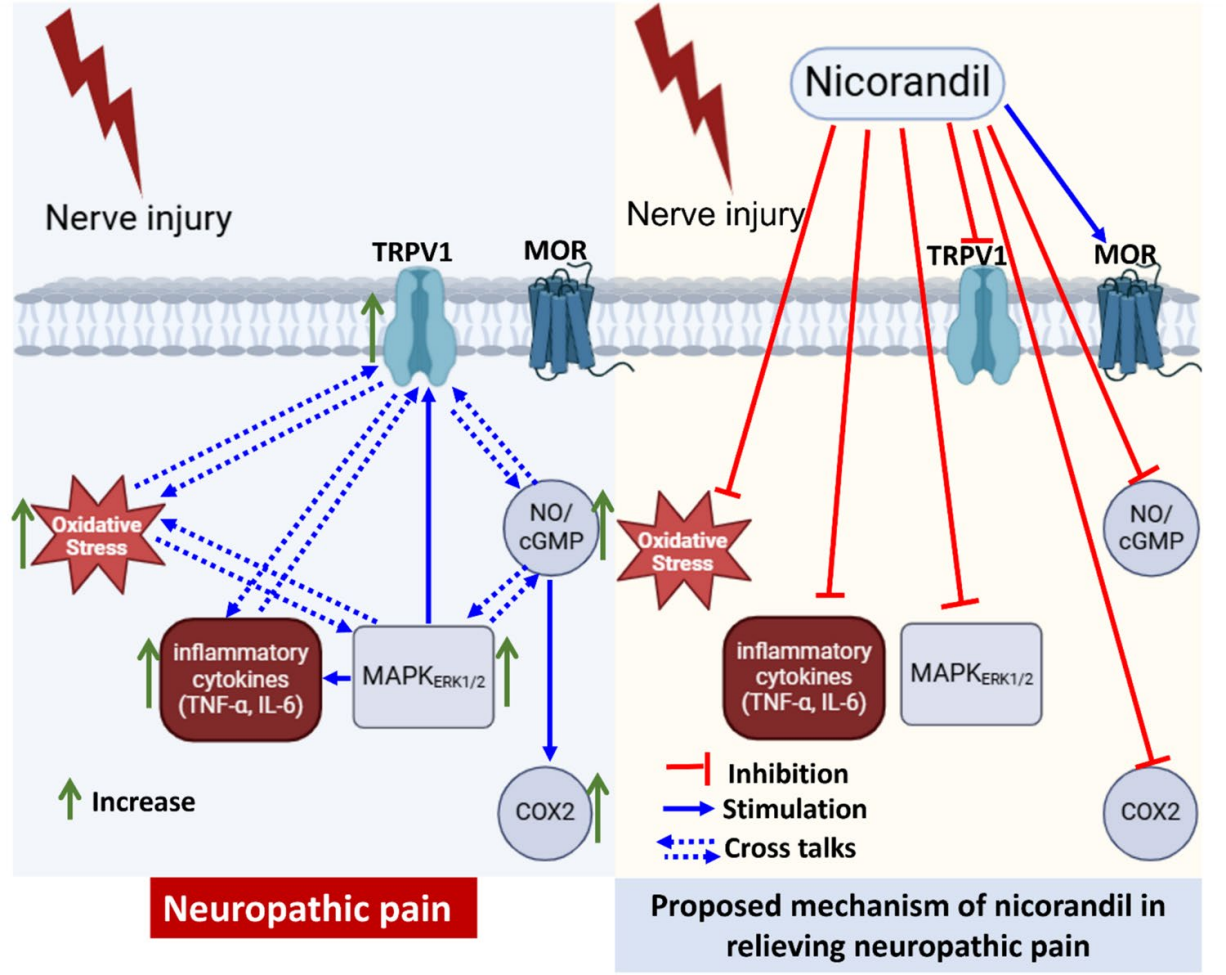
Proposed mechanism of nicorandil in relieving neuropathic pain. TRPV1, transient receptor potential vanilloid 1; MOR, µ opioid receptor; NO, nitric oxide; cGMP; cyclic guanosine monophosphate; TNF-α, tumor necrosis factor alpha; IL-6, interleukin 6; MAPK_ERK1/2_; mitogen activated protein kinase (extracellular signal-regulated kinase 1/2); COX2, Cyclooxygenase2.

Collectively, multiple mechanisms are implicated in nicorandil antinociception in neuropathic and nociceptive pain. Nicorandil antinociception is due to direct modulation of NO/cGMP signaling, and indirectly; presumably, via inhibition of oxidative mediators/inflammatory cytokines, MAPK_ERK1/2_ and TRPV1 receptors. The findings suggest that nicorandil could serve as an alternative option for managing neuropathic pain due to its multi-targeted mechanisms. A proposed mechanism of nicorandil in relieving neuropathic pain is illustrated in Fig. 13. Together, the results emphasize that nicorandil should be further explored aiming its readjusting in the treatment of various painful disorders.

## Data Availability

Data analyzed during the current study are available from the corresponding author on reasonable request.

## Abbreviations

AUC: Area under the curve
CNS: Central nervous system
CCI: Chronic constriction injury
cGMP: Cyclic guanosine monophosphate
COX-2: Cyclooxygenase-2
CMC: Carboxymethyl cellulose
DMSO: Dimethyl sulfoxide
DRG: Dorsal root ganglion
ELISA: Enzyme-linked immunosorbent assay
ERK1/2: Extracellular signal-regulated kinase 1/2
GC: Guanylyl cyclase
H&E: Hematoxylin and eosin
IASP: International Association for the Study of Pain
IL-6: Interleukin-6
i.pl.: Intraplantar
I.P.: Intraperitoneal
IV: Intravenous
K_ATP_: ATP-sensitive potassium channel
L-NAME: Nω-nitro-L-arginine methyl ester
MAPK: Mitogen-activated protein kinase
MB: Methylene blue
MDA: Malondialdehyde
nNOS: Neuronal nitric oxide synthase
NO: Nitric oxide
ODQ: 1H-oxadiazolo[3-a]quinoxalin-1-one
PO: Per os (oral administration)
PKG: Protein kinase G
ROS: Reactive oxygen species
s.c.: Subcutaneous
TNF-α: Tumor necrosis factor-alpha
TRPV1: Transient receptor potential vanilloid 1
